# Multiscale anisotropy analysis of second-harmonic generation collagen imaging of human pancreatic cancer

**DOI:** 10.3389/fonc.2022.991850

**Published:** 2022-10-18

**Authors:** Joshua Hamilton, Anne Breggia, Timothy L. Fitzgerald, Michael A. Jones, Peter C. Brooks, Karissa Tilbury, Andre Khalil

**Affiliations:** ^1^ Chemical and Biomedical Engineering, University of Maine, Orono, ME, United States; ^2^ CompuMAINE Laboratory University of Maine, Orono, ME, United States; ^3^ Graduate School of Biomedical Sciences and Engineering, University of Maine, Orono, ME, United States; ^4^ Center for Applied Science and Technology, Maine Health Institute for Research, Scarborough, ME, United States; ^5^ Department of Surgical Oncology, Maine Medical Center, Portland, ME, United States; ^6^ Spectrum Healthcare Partners, Portland, ME, United States; ^7^ Center for Molecular Medicine, MaineHealth Institute for Research, Scarborough, ME, United States

**Keywords:** anisotropy, second-harmonic generation (SHG), pancreatic cancer, wavelets, tumor micro environment

## Abstract

Pancreatic ductal adenocarcinoma (PDAC) is one of the deadliest cancers with a minority (< 10%) of patients surviving five years past diagnosis. This could be improved with the development of new imaging modalities for early differentiation of benign and cancerous fibrosis. This study intends to explore the application of a two-photon microscopy technique known as second harmonic generation to PDAC using the 2D Wavelet Transform Modulus Maxima (WTMM) Anisotropy method to quantify collagen organization in fibrotic pancreatic tissue. Forty slides from PDAC patients were obtained and eight images were captured per each tissue category on each slide. Brownian surface motion and white noise images were generated for calibration and testing of a new variable binning approach to the 2D WTMM Anisotropy method. The variable binning method had greater resistance to wavelet scaling effects and white noise images were found to have the lowest anisotropy factor. Cancer and fibrosis had greater anisotropy factors (Fa) at small wavelet scales than normal and normal adjacent tissue. At a larger scale of 21 μm this relationship changed with normal tissue having a higher Fa than all other tissue groups. White noise is the best representative image for isotropy and the 2D WTMM anisotropy method is sensitive to changes induced in collagen by PDAC.

## Introduction

Pancreatic cancer is the deadliest solid organ malignancy, the 11^th^ most common cancer but the 3^rd^ most common cause of cancer-related death (1). At the time of presentation majority of patients have either locally advanced or metastatic disease; this presentation has a median survival of fewer than six months. Despite novel combinations of multiagent chemotherapy, this disease is fatal in most patients (2, 3). Tumor-associated stroma and microenvironment may provide an immunoprotective milieu; the fibrotic nature of PDAC frequently limits both chemotherapy and immunotherapy penetration (4–6). Unfortunately, conventional imaging modalities poorly differentiate benign fibrosis from cancerous fibrosis (7–9). Therefore, proper identification of cancerous fibrosis is critical for treatment decisions.

To develop a biologically meaningful diagnostic or treatment method, the underlying markers, and biophysical properties of PDAC development need to be better understood. Stromal cell heterogeneity leads to genetic predisposition to chemo and immunotherapy resistance in multiple cancers (10, 11). This heterogeneity is also tied to the severity of fibrosis caused by PDAC in the pancreas (12). Higher levels of fibrosis and stromal stiffness are also correlated with poor patient prognosis (13). Attempts to understand this relationship involved the use of PDAC mouse models in which researchers inhibited the ability of stroma cells to produce extracellular matrix (ECM) components such as collagen (14). When compared to the control PDAC models, the inhibition of collagen increased tumor cell metastasis and lethality (14). This suggests that the relationship between the activation of stromal cells, fibrosis, and cancer severity are more complex than a simple linear correlation. The remodeling of the extracellular matrix in the tumor microenvironment is a complex interaction between the PDAC cells and stromal cells, that orchestrates the loss of homeostasis with respect to collagen synthesis, degradation, and post-translational modification (15). Collagen in PDAC is typically longer, straighter, and more aligned than healthy pancreatic tissue collagen (16–18). Different structures of collagen have been shown to interact with T cell penetration leading to poor patient outcomes (6). One hypothesis is that stromal cell heterogeneity impacts the dynamics of collagen remodeling promoting tumorigenesis and suppressing immune system penetration into the tumor mass (19).

Exploration of the relationship between collagen and cancer has been conducted in a multitude of studies using Second Harmonic Generation (SHG) (20). SHG is a two-photon microscopy technique that uses pulsed lasers to elicit a non-linear response in the tissue (21). SHG microscopy is a label-free, collagen specific imaging technique with submicron resolution and 3D optical sectioning (22–24). The typical excitation wavelength used for collagen is in the infrared (IR) range and capable of penetrating tissue upwards of 500 μm (25). This would allow for imaging PDAC tumor biopsies with minimal modification to current clinical approaches. Recent studies applying SHG to PDAC use an open software known as CT-FIRE to quantify collagen fiber morphology (16–18, 20). First, a fixed-size curvelet is convolved with the image and then a fiber-tracing algorithm delineates individual collagen fibers to quantify collagen fiber width, length, alignment, and straightness. CT-FIRE is focused on identifying characteristics of single collagen fibers at a single scale; however, understanding the overall alignment at multiple convolution scales could be the key to a deeper understanding of PDAC ECM modification.

A recent publication highlighted the need for a multiscale approach to understanding collagen fiber organization in biological systems using the 2D Wavelet Transform Modulus Maxima (WTMM) anisotropy method on SHG images (26). The 2D WTMM anisotropy method involves convolving the image with Gaussian derivative wavelets over a continuous range of smoothing scales enabling a multiscale approach to understand organizational changes in an image. The method was first developed in the context of Galactic astronomy (27) and later used in a multitude of applications such as muscle morphogenesis (28–32), artificial bone implants (33), exploring the relationship between nerve and college in neuropathic adipose tissue  (34), and the aforementioned exploration into comparing mouse melanoma and integrin knockout impact on collagen restructuring (26). To optimize this method for subtle changes between cancer and fibrotic collagen, updates to the 2D WTMM anisotropy method are being explored and presented in this paper.

This paper builds upon the previously published multiscale 2D WTMM anisotropy method to increase the sensitivity of the final anisotropy factor with the introduction of isotropic calibration images and statistically-driven histogram binning processes. This improved method is applied to SHG images of PDAC pathology slides annotated by a pathologist to reveal multiscale alterations of collagen fiber morphologies. This once again demonstrates the need of multiscale approaches to understanding the ECM, especially due to disease state alterations.

## Materials and methods

### Pathology slides

Forty H&E stained slides of PDAC tissue, from forty unique patients, originally biopsied for pathology analysis were obtained from the Maine Medical Center BioBank (MMC BB). The slides were scanned with an Aperio2 slide scanner (Leica Biosystems, Wetzlar, Germany) and then annotated by a pathologist who highlighted areas of cancer, fibrosis, and normal tissue. Twenty slides were annotated as only normal tissue and the other twenty slides had areas annotated as cancer. Of the twenty cancer slides, 15 also had areas annotated as fibrosis and 13 had areas annotated as normal tissue. Normal tissue on the same slide as cancer slide were categorized as normal adjacent tissue. This results in four tissue categories from the annotated slides, cancer, normal, fibrosis, and normal adjacent (see **Table 1**).

**Table 1:** Summary of pancreatic biopsy slide information by patient.

Patient Deidentified Label	TNM Classification^1^	Grade of Tumor	Location of PDAC in Pancreas	Slide is Normal Only Tissue(Normal)	Tissue Labelled as Cancer(Cancer)	Tissue Labelled as Fibrotic(Fibrosis)	Tissue Labelled as Normal(Normal Adjacent)
R14-0077	pT3, N1	3	Head	No	Yes	Yes	Yes
R14-0370	pT3, pN0	3	Tail	No	Yes	Yes	No
R16-0393	pT3, pN1, pMX	2	Tail	No	Yes	No	No
R16-0596	pT3, pN0	2	Head	No	Yes	Yes	Yes
R16-0642	ypT3, ypN1	2	Tail	No	Yes	Yes	No
R16-0975	pT3, pN1	3	Tail	No	Yes	Yes	No
R16-1024	ypT3, ypN1	2	Head	No	Yes	Yes	No
R16-1309	ypT3, ypN1	3	Head	No	Yes	Yes	No
R17-0139	ypT3, ypN0	2	Head	No	Yes	Yes	Yes
R17-0206	pT4, pN0	3	Body & Tail	No	Yes	Yes	Yes
R17-0293	pT3, pN1, pMX	2	Head	No	Yes	No	Yes
R17-0339	ypT2, ypN0	3	Head	No	Yes	Yes	No
R17-0404	pT3, pN0	2	Head	No	Yes	Yes	Yes
RA01-1347	T3, pN1a, Mx	3	Head	No	Yes	Yes	Yes
RA02-3061	T3, pN1b, Mx	2	Head	No	Yes	Yes	Yes
RA03-0972	T3	3	Head	No	Yes	No	Yes
RA03-1977	T3, N1, MX	2	Head	No	Yes	Yes	Yes
RA03-2818	T1b, N0, MX	2	Head	No	Yes	No	Yes
RA03-3112	T3, N1, MX	3	Head	No	Yes	Yes	Yes
RA99-3459	T3, N1, MX	3	Head	No	Yes	No	Yes
R11-0633	ypT3, ypN0, ypMX	2	Body	Yes	N/A	N/A	N/A
R15-0397	pT3, pN0	1	Body & Tail	Yes	N/A	N/A	N/A
R16-0063	ypT3, ypN1	2	Head	Yes	N/A	N/A	N/A
R16-0194	ypT3, ypN1	2	Head	Yes	N/A	N/A	N/A
R16-0756	pT3, pN0, pMX	3	Head	Yes	N/A	N/A	N/A
R16-0848	pT3, pN0	1	Head	Yes	N/A	N/A	N/A
R16-0931	ypT3, ypN0	2	Head	Yes	N/A	N/A	N/A
R16-0975	pT3, pN1	3	Tail	Yes	N/A	N/A	N/A
R16-1182	pT1, pN1	2	Head	Yes	N/A	N/A	N/A
R16-1302	ypT3, ypN0	2	Head	Yes	N/A	N/A	N/A
R17-0139	ypT3, ypN0	2	Head	Yes	N/A	N/A	N/A
R17-0293	pT3, pN1, pMX	2	Head	Yes	N/A	N/A	N/A
R17-0339	ypT2, ypN0	3	Head	Yes	N/A	N/A	N/A
RA01-1608	T3, pN1a, MX.	3	Head	Yes	N/A	N/A	N/A
RA03-3169	T2, N1, MX	3	Head	Yes	N/A	N/A	N/A
RA04-1875	T3, N0, MX	2	Body	Yes	N/A	N/A	N/A
RA05-0043	T3, N1, MX	3	Head	Yes	N/A	N/A	N/A
RA06-0840	T3, N1, MM	3	Head	Yes	N/A	N/A	N/A
RA06-0866	T1, N0, MX	2	Tail	Yes	N/A	N/A	N/A
RA06-0891	pT3, pN1b, MX	3	Head	Yes	N/A	N/A	N/A
**Summary:**	N/A	N/A	N/A	20	20	15	13
**# of Images:**	N/A	N/A	N/A	160	160	120	104

N/A, Not Applicable.

### Imaging

**Figure 1:**
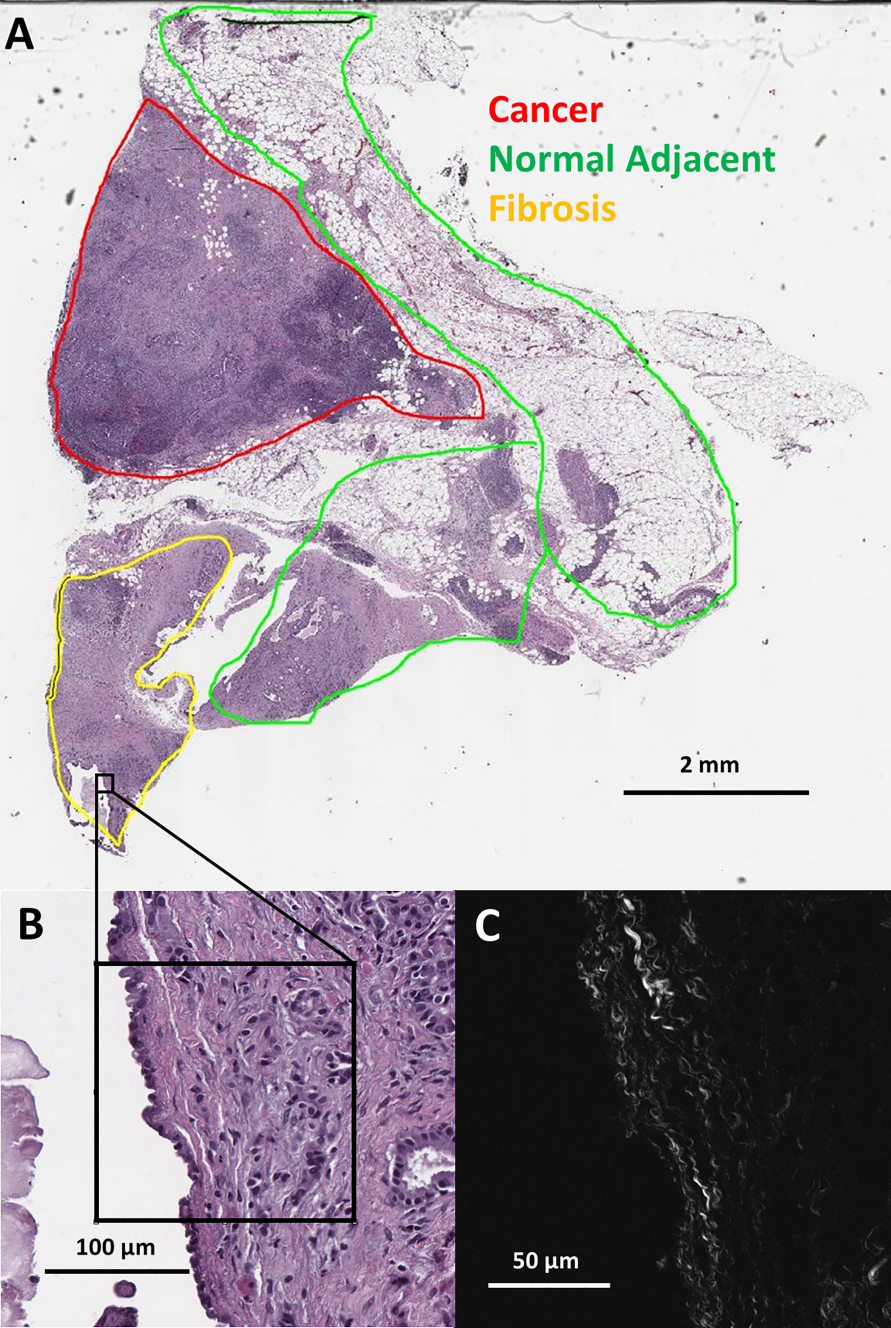
**(A)** Pathologist labeled H&E slide scan from a pancreatic tumor biopsy with an example of one SHG microscopy imaged area outlined in black. **(B)** Zoomed view of outlined area from H&E scan. **(C)** SHG microscopy image of outlined area.

Eight distinct ductal structures within each normal, normal adjacent, fibrosis, and cancer were identified on the H&E slide and then imaged using SHG microscopy (**Figure 1**). SHG images were acquired on a custom-built 2-photon microscope consisting of an upright microscope stand (Olympus BX50WI, Olympus, Center Valley, Pennsylvania), laser scanning unit (Fluoview300, Olympus), titanium sapphire femtosecond laser (Chameleon Ultra II, Coherent, Santa Barbara, California), and an electro optic modulator (ConOptics, Danbury, Connecticut) for laser power modulation. Circular polarization was used for SHG imaging; it was verified at the focal plane by rotating a polarizer and experiencing no change in laser power. Forward directed SHG images were acquired at 890-nm excitation, using a LUMPlanFLN 40 × 0.8 NA (Olympus, Center Valley, Pennsylvania) water immersion objective. Forward directed SHG was collected in a 0.9 NA condenser lens and filtered using a 448/20 bandpass filter and the forward directed SHG signal was collected using a 448/20-nm (Semrock Rochester, New York) prior to detection *via* a H7421 GaAsP PMT (Hamamastsu, Hamamstsu City, Japan). Each region of interest was imaged using 2x optical zoom (180 μm field of view) with a digital resolution of 512 × 512 pixels and using a laser scanning speed of 2.71 s/frame with the final image resulting from Kalman filtering using 4 state updates. The full list of patients, images, and tissue categories used in this study are shown in **Table 1**.

### The 2D WTMM anisotropy method

The SHG images were analyzed using the 2D WTMM anisotropy method. This method involves convolving the image with wavelets over a continuous range of scales allowing for multiscale analysis. The wavelets used for the analysis are the partial derivatives of a 2D Gaussian (isotropic) smoothing function, *ϕ*(*x*,*y*) :


(1) $$\psi_{1}(x,y)=\;\frac{\partial \phi (x,y)}{\partial x}\text{ and }\psi_{2}(x,y)=\;\frac{\partial \phi (x,y)}{\partial y}$$

The 2D wavelet-transform applied to the image, *f*, is calculated as follows:


(2) $$T_{\psi }[f](b,a)=\left(\begin{array}{c} T_{\psi_{1}}[f]\;=\;a^{-2}\int^{}d^{2}x\psi_{1}(a^{-1}(x–b))f(x)\\ T_{\psi_{2}}[f]\;=\;a^{-2}\int^{}d^{2}x\psi_{2}(a^{-1}(x–b))f(x) \end{array}\right)$$

$$=T_{\psi }[f](b,a)=\;\nabla \left\{T_{\phi }[f](b,a)\right\}$$

from which the modulus and argument (angle) can be obtained:


(3) $$M_{\psi }[f](b,a)={\left[{\left(T_{\psi_{1}}[f](b,a)\right)}^{2}+{\left(T_{\psi_{2}}[f](b,a)\right)}^{2}\right]}^{1/2}$$

$$A_{\psi }[f](b,a)=\text{ Arg}\left(T_{\psi_{1}}[f](b,a)+iT_{\psi_{2}}[f](b,a)\right)$$

**Figure 2:**
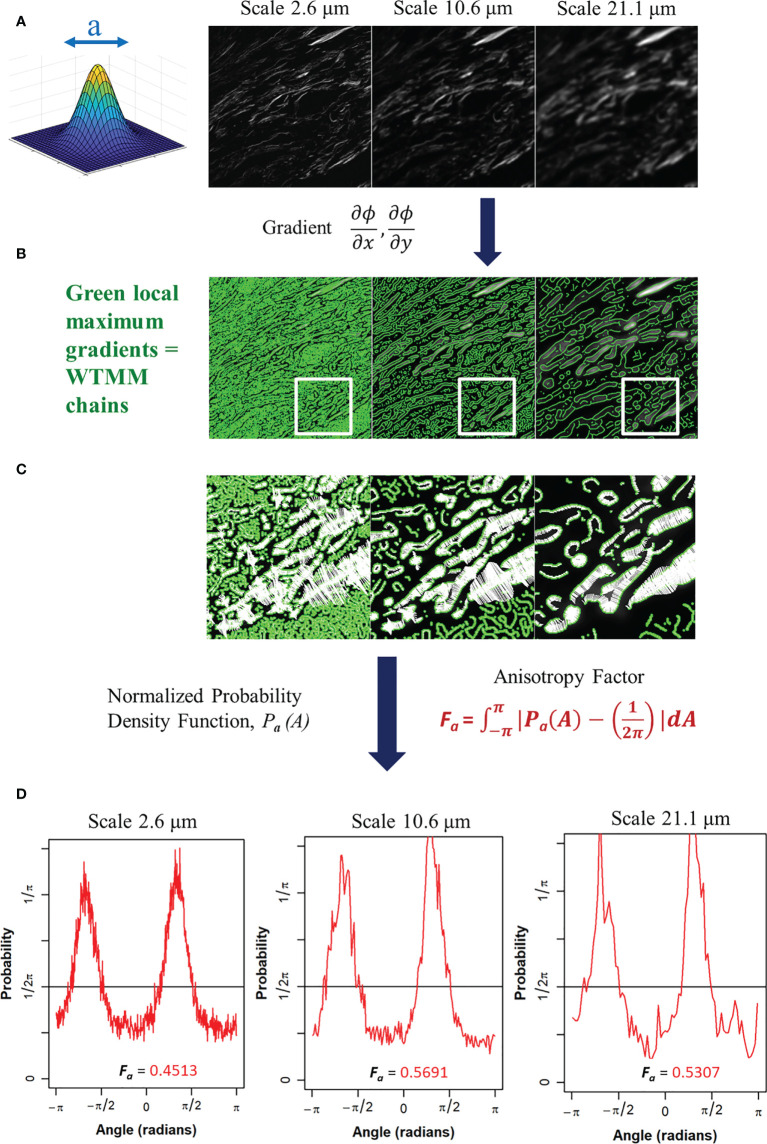
**(A)** Gaussian wavelet convolved SHG image at 3 representative wavelet size scales. **(B)** Maxima chains at 3 representative wavelet scales shown in green. **(C)** Zoomed inserts from white outlined area in panel B at 3 representative wavelet size scales to identify WTMM vectors (white arrows) showing magnitude and direction of local gradients of the maximum image density fluctuations. **(D)** Probability density functions (PDF), shown in red, of 3 representative wavelet scales generated from angles of local gradient vectors with the theoretical isotropic 1/2pi line shown in black. The anisotropy factor, Fa (Eq. 4), is shown for each PDF.

At a given scale *a* > 0, the wavelet transform modulus maxima are the specific locations **b** within the image where the modulus *M_ѱ_* [*f*] (**b**,*a*) is locally maximum in the direction of the argument *A_ѱ_* [*f*] (**b**,*a*). At each size scale *a*, these WTMM are automatically organized as “edge detection maxima chains” (35). Additional algorithmic details can be found in the Appendix of reference (36). A visual diagram of these mathematical operations is shown in **Figure 2**.

The angles from the WTMM chains are used to find the direction of changes in the image intensity. This allows for a definition of anisotropy based off the directional changes of image intensity across all size scales *a*. The angle distribution from the WTMM chains at each size scale are used to calculate probability density functions (PDFs), *P_a_* (*A*), of the angles. A flat PDF indicates a perfectly isotropic image with random direction of intensity variations. Therefore, deviations from a flat PDF are indicative of intensity variations at preferential angles (anisotropy). Numerically, binning of the PDF is typically fixed to a single number of histogram bins (i.e. 64) at each size scale regardless of the total number of maxima chains (26). Here, there are significant differences in the number of maxima chains at each scale; therefore, a variable binning approach was developed and implemented. In this variable binning approach, each scale has a unique number of bins to ensure 100 maxima chains are in each bin. To generate a quantitative measurement from the wavelet-transformed image at each size scale we used the anisotropy factor, *F_a_* where at each size scale *a*, the area between the curve of the image pdfs and a flat, $\frac{1}{2\pi }$ , pdf is calculated:


(4) $$F_{a}=\;\int_{-\pi }^{\pi }\left|P_{a}(A)-\frac{1}{2\pi }\right|dA\approx \;\Sigma_{i=0}^{{\text{N}}_{bins}}\left|P_{a}(A_{i})-\frac{1}{2\pi }\right|\Delta A\;\;$$

$$where\;\Delta A=\frac{2\pi }{{\text{N}}_{bins}}\;,\;A_{0}=\;-\pi ,\;A_{i}=A_{0}+\Delta A\cdot i\;$$

A value of *F_a_* = 0 represents pure isotropy and greater values represent more anisotropy where the theoretical upper limit is 2 (See **Supplemental Data** for Details). This process is also shown in **Figure 2**. This results in a quantitative representation of image anisotropy which in turn provides information on collagen morphology in this study.

### Generation of Brownian motion surface and white noise images

For numerical calibration of isotropy, two variations of self-affined, scale invariant fractal images were used: Brownian motion surfaces and white noise images. Both are known to be theoretically isotropic, i.e., their pixel intensity gradients change randomly (37). Brownian motion (136) and white noise (136) images were generated using the Fourier filtering method (37) to be studied using the 2D WTMM anisotropy method.

### Numerical implementation

The numerical calculations described in Sections 2.3 and 2.4 were performed using Xsmurf, a Tcl/Tk software package that runs C-based routines (github.com/pkestene/xsmurf). The statistical analyses were performed using the R package (38).

## Results

### White noise vs. Brownian

**Figure 3:**
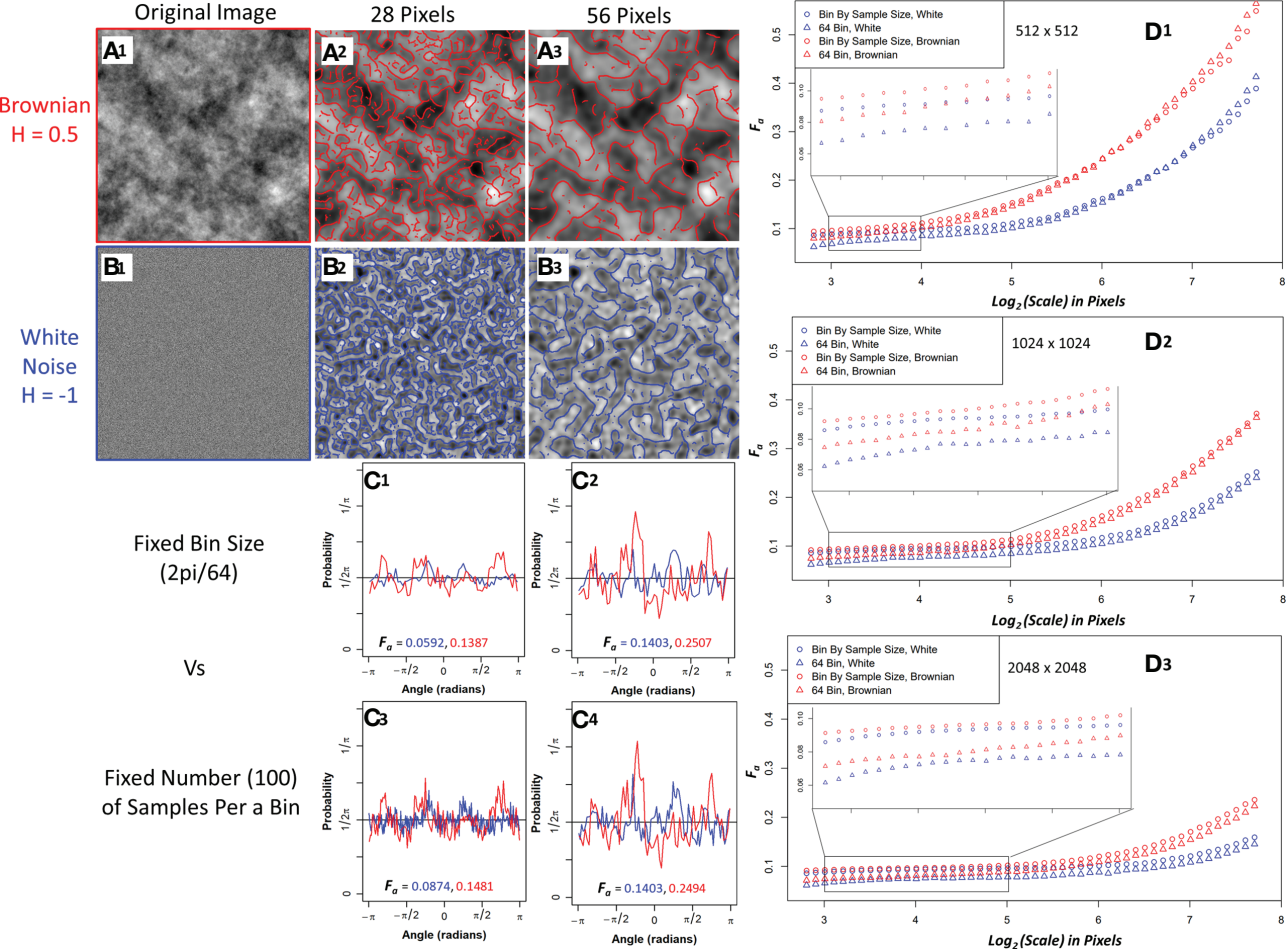
Sample Brownian image **(A1)** and sample white noise image **(B1)** convolved with a 28-pixel wavelet **(A2, B2)** and a 56-pixel wavelet **(A3, B3)** with maxima chains shown in red (Brownian) and blue (white noise). Probability density functions (PDF) with anisotropy factors from two representative size scales using 64 bins to generate the PDF **(C1, C2)** and fixing the number of samples (100) per a bin to generate the PDF **(C3, C4)**. Brownian is shown in red and white noise in blue. Median anisotropy factor vs wavelet scale across 136 images for Brownian (red) and white noise (blue) 512 x 512-pixel images **(D1)**, 1024 x 1024-pixel images **(D2)**, and 2048 x 2048-pixel images **(D3)** with fixed 64 binning for anisotropy factor calculation (Triangle) and 100 samples per a bin calculation (Circle). Inset graph is a zoomed in view of anisotropy factor vs scale within the enclosed region.

The white noise and Brownian motion surface image analysis was computed using the 2D WTMM anisotropy method described earlier. The images were analyze using fixed binning (64 bins) and variable binning (100 samples a bin) with the results shown in **Figure 3**. Variable binning causes significant changes in the pdf and anisotropy factor at small scales but causes minimal changes at larger scales (**Figure 3C1-C4**). Furthermore, the anisotropy factor vs wavelet scale is more stable with variable binning in both Brownian motion surface and White noise images which is ideal due to their known scale invariance (**Figure 3D1-D3**, zoomed inserts). Additionally, the white noise curves at all image size scales have a lower anisotropy factor than the Brownian motion surface images. The white noise curves also remain flat for a longer wavelet scale range suggesting more robust scale invariance.

To further investigate their differences, edge effects on both image types were assessed by calculating the wavelet transform on full 1024x1024 and 2048x2048 images and then only using the center 512x512 maxima chains for anisotropy analysis. White noise images had less anisotropy factor variability when compared to the actual 512x512 images than Brownian motion surface images, thus suggesting white noise images are more resistant to edge effects (**Supplemental Figure 1**). Variable binning was also found to improve the coefficient of variation in the anisotropy factor of white noise images at all size scales, with a significant improvement at smaller size scales (**Supplemental Figure 2**). Given the superiority of white noise and variable binning, the anisotropy factor of the pancreatic images were variably binned with white noise images used as the numerical isotropic control.

### PDAC SHG WTMM anisotropy analysis results

**Figure 4:**
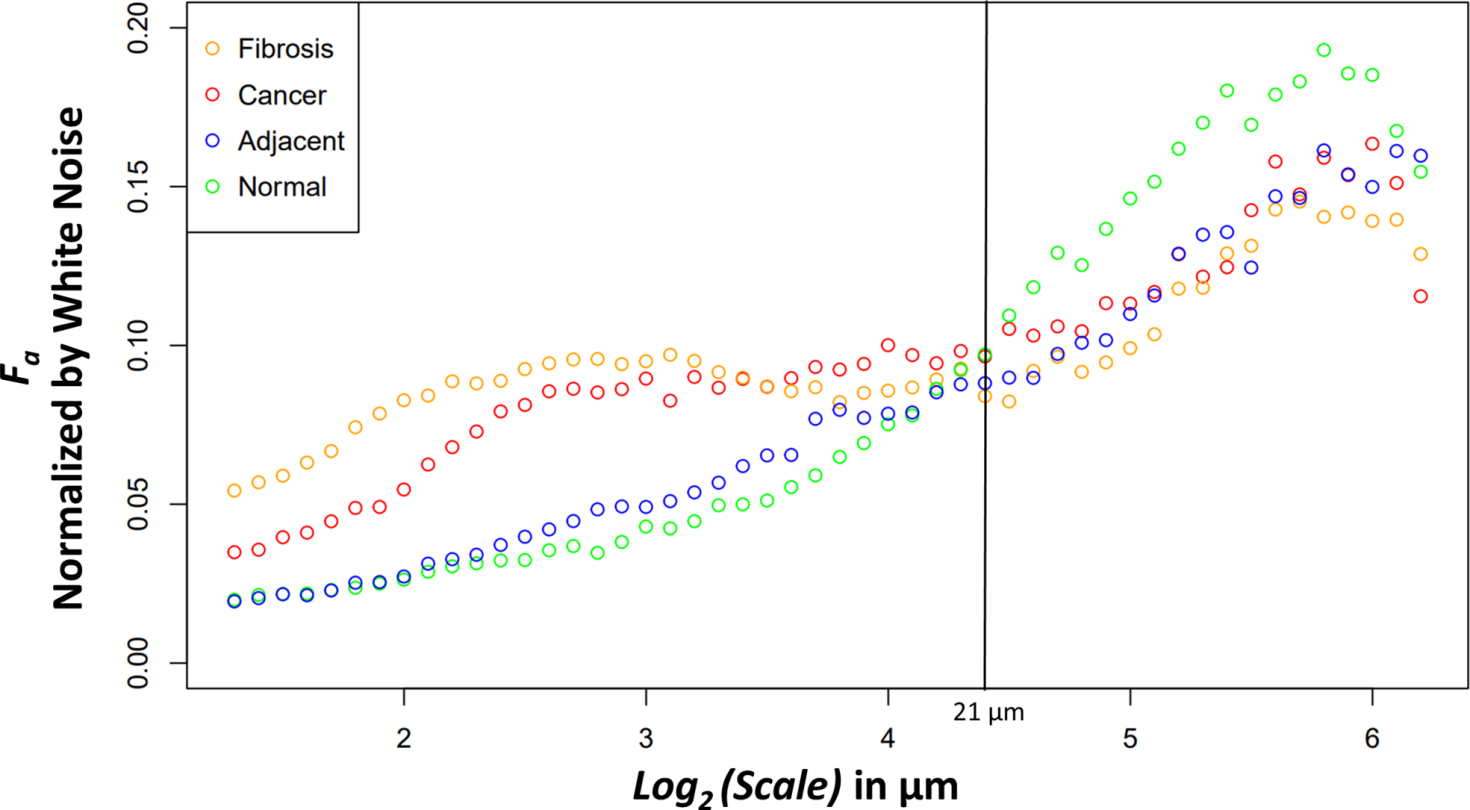
Median anisotropy factor across all images for fibrosis (orange), cancer (red), adjacent (blue), and normal (green) tissue normalized by white noise anisotropy factor from ~2.5 μm to 73.5 μm.

The new variable binned, white noise controlled, multi-scale anisotropy factor results of normal, normal adjacent, fibrosis, and cancer are plotted in **Figure 4**. There are significant differences in anisotropy factor values as can be seen between the cancer/fibrosis and normal/adjacent categories. Interestingly, at a wavelet scale of 21 µm all tissue types converge with only normal tissue diverging at larger scales.

**Figure 5:**
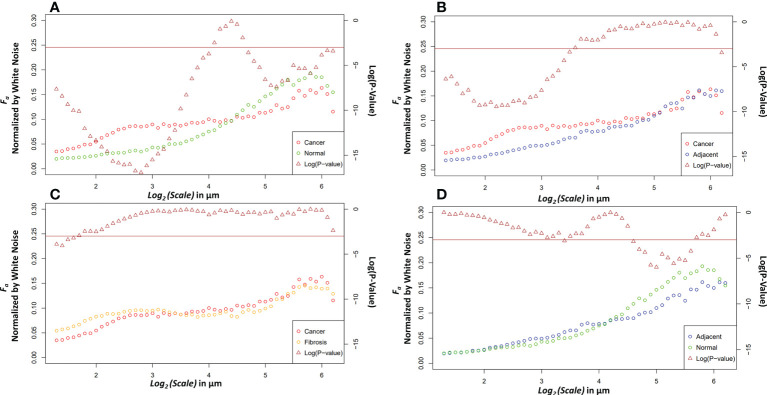
Pairwise comparisons of the median anisotropy factor across all images normalized by white noise anisotropy factor from ~2.5 μm to 73.5 μm (left *y*-axis) for cancer tissue *vs.* normal tissue **(A)**, cancer tissue *vs*. adjacent tissue **(B)**, cancer tissue *vs.* fibrotic tissue **(C)**, and adjacent tissue *vs.* normal tissue **(D)**. The *p*-values (right *y*-axis), shown in brown, from a Wilcoxon rank sum test ran at each scale between the tissue pair’s anisotropy factors. Values below the horizontal brown line at 0.05 show statistical significance.

Four relevant pairwise comparisons are shown in **Figure 5** with corresponding Wilcoxon ranked sum test. Cancer vs normal, shown in panel A, are statistically significantly different at all size scales other than the convergence wavelet range. At smaller size scales, before the cross over scale, cancer is more anisotropic and at larger size scales, after the cross over scale, the normal tissue is more anisotropic. In Panel B, the comparison between adjacent and cancer tissue can be seen where they are statistically significantly different at small wavelet size scales but are not at the larger wavelet size scales.

The fibrosis and cancer are not statistically significantly different except at the first four wavelet size scales shown in panel C. In panel D, the normal tissue is more anisotropic than normal adjacent tissue at larger size scales but is not statistically significantly different at other size scales.

## Discussion and conclusions

This paper shares improvements made to the 2D WTMM anisotropy method, and an exploration of the method applied to PDAC pathology slides. The method improvements help define isotropy vs. anisotropy within the 2D WTMM anisotropy method and better utilize the statistics at smaller scales through variable binning as demonstrated by the improvement in coefficient of variation. The statistical significance found between tissue labeled as cancerous or as normal by a pathologist demonstrate the future possible clinical applications of this label-free analysis method.

### White noise and Brownian motion surface discussion

The 2D WTMM anisotropy method testing performed on the two scale invariant normalization candidates tell a clear story. In every test the white noise outperformed the Brownian motion surface images demonstrating white noise as the clear candidate for discrete numerical normalization of isotropy. This result follows the logic that can be derived about the image structure due to its generation. The Brownian motion surface images have large-scale sinusoidal patterns as they move from high to low pixel value areas in the image. The 2D WTMM anisotropy method at larger scales detects this large-scale sinusoidal pattern, which then results in the higher anisotropy factor than white noise. At smaller scales, the Brownian surface motion image could have pockets of pixels that seem more anisotropic from its gradual pixel value changes whereas white noise is isotropic at all scales.

### Biological interpretation

The differences between the tissue types can elucidate information about ECM modification in both close and distant proximity from the tumor. The higher anisotropy of cancer at smaller scales could be caused through stellate cell over production of collagen and cancer cell mediated remodeling (39). At larger scales, after an interesting ~21 μm wavelet size crossover point, the larger anisotropy in healthy tissue suggests that the cancer has disrupted the overall normal large-scale organization of collagen fiber bundles. This observation is further supported by the adjacent normal tissue results. The cancer and adjacent normal tissue are different at smaller scales, but this statistical significance starts dissipating at a wavelet scale of ~12 μm. At larger size scales the adjacent tissue cannot be differentiated from cancer suggesting again a large-scale collagen disruption due to the cancer even in areas distant from those annotated as cancer by a pathologist. An important note when comparing normal and adjacent normal tissue is the lack of 3D spatial organization as this study was prospective and careful 3D mapping of pancreatic tissue processing was not performed. Therefore, it is plausible that a slide containing only normal tissue is near-adjacent to cancerous tissues in a manner similar to normal adjacent tissue on a slide annotated with both cancerous and normal tissue. Despite this possibility, there was statistical significance between normal adjacent and normal tissues at larger scales suggesting that collagen organization is disrupted near cancerous tissue. Finally, the comparison of fibrosis vs cancer only found slight statistical significance over the first four wavelet scales which range from ~2.4 μm to ~3.0 μm. This difference could be due to the straightness of the fibers in the fibrotic tissue. Otherwise, the overall trend of anisotropy factor vs scale matched between the two tissue types. This suggests that that there are minor differences in collagen structure between fibrosis and the cancer collagen. The pathologist labeled these tissue types using the gold standard, H&E histology, but it is highly subjective, and moreover, not highly sensitive to the collagen fibrillar aspects which seem to contain some valuable information regarding normal vs cancerous vs fibrotic tissue. Overall, this method demonstrates the ability to differentiate changes in collagen structure induced by PDAC using a continuous multiscale suite of information allowing for deeper analysis than other methods.

## Data availability statement

The image data supporting the conclusions of this article will be made available by the authors, without undue reservation.

## Ethics statement

Ethical review and approval was not required for the study on human participants in accordance with the local legislation and institutional requirements. Written informed consent for participation was not required for this study in accordance with the national legislation and the institutional requirements.

## Author contributions

AB, TF, MJ, PB: clinical data acquisition. JH: image pre-processing. JH and AK: WTMM analysis. JH, KT, and AK: statistical analyses and figure preparation. JH, AB, PB, TF, KT, and AK: manuscript writing. All authors contributed to the article and approved the submitted version.

## Funding

We acknowledge support from the Jane Bellino Cancer Research Fund, administered through the Maine Medical Center Philanthropy Department.

## Acknowledgments

We are thankful to Dr. Thomas Gridley and all of the CompuMAINE Lab members for helpful technical discussions.

## Conflict of interest

The authors declare that the research was conducted in the absence of any commercial or financial relationships that could be construed as a potential conflict of interest.

## Publisher’s note

All claims expressed in this article are solely those of the authors and do not necessarily represent those of their affiliated organizations, or those of the publisher, the editors and the reviewers. Any product that may be evaluated in this article, or claim that may be made by its manufacturer, is not guaranteed or endorsed by the publisher.

## Footnotes

1
https://www.cancerresearchuk.org/about-cancer/pancreatic-cancer/stages-types-grades/tnm-staging.
