# Supplementary material for: Multiscale anisotropy analysis of second-harmonic generation collagen imaging of human pancreatic cancer
Source: Front Oncol. 2022 Oct 18;12:991850. doi: 10.3389/fonc.2022.991850 (PMC9623060; doi:10.3389/fonc.2022.991850)
Supplement: Supplementary file 1 [file DataSheet_1.docx]

Supplementary Material

# Supplementary Data

## Mathematical Proof of Anisotropy Factor Upper Limit

$$For any scale>0, P_{a}\left( A \right) \left( \mathrm{where} A \in\left[ -\pi, \pi\right] is an angle in radians \right) is a normalized$$

$$probability distribution function, i.e.$$

$\int_{-\pi}^{\pi} P_{a}\left( A \right)dA=1$ (1)

$$The anisotropy factor, F_{a}, is defined as$$

$F_{a}= \int_{-\pi}^{\pi} |P_{a}\left( A \right)-\frac{1}{2\pi}|dA$ (2)

$$and the theoretical upper bound of F_{a} can be determined using this conservative inequality$$

$\left| x-y \right|\leq\left| x \right|+\left| y \right|.$ (3)

$$When the inequality is applied to F_{a}, it follows as shown,$$

$F_{a}= \int_{-\pi}^{\pi} |P_{a}\left( A \right)-\frac{1}{2\pi}|dA \leq\int_{-\pi}^{\pi} \left( \left| P_{a} \right|+\left| \frac{1}{2\pi} \right| \right)dA$

$\leq\int_{-\pi}^{\pi} P_{a}\left( A \right)dA+\int_{-\pi}^{\pi} \left| \frac{1}{2\pi} \right|dA$ (4)

$$= 1+1=2.$$

# Supplementary Figures and Tables

## Supplementary Figures


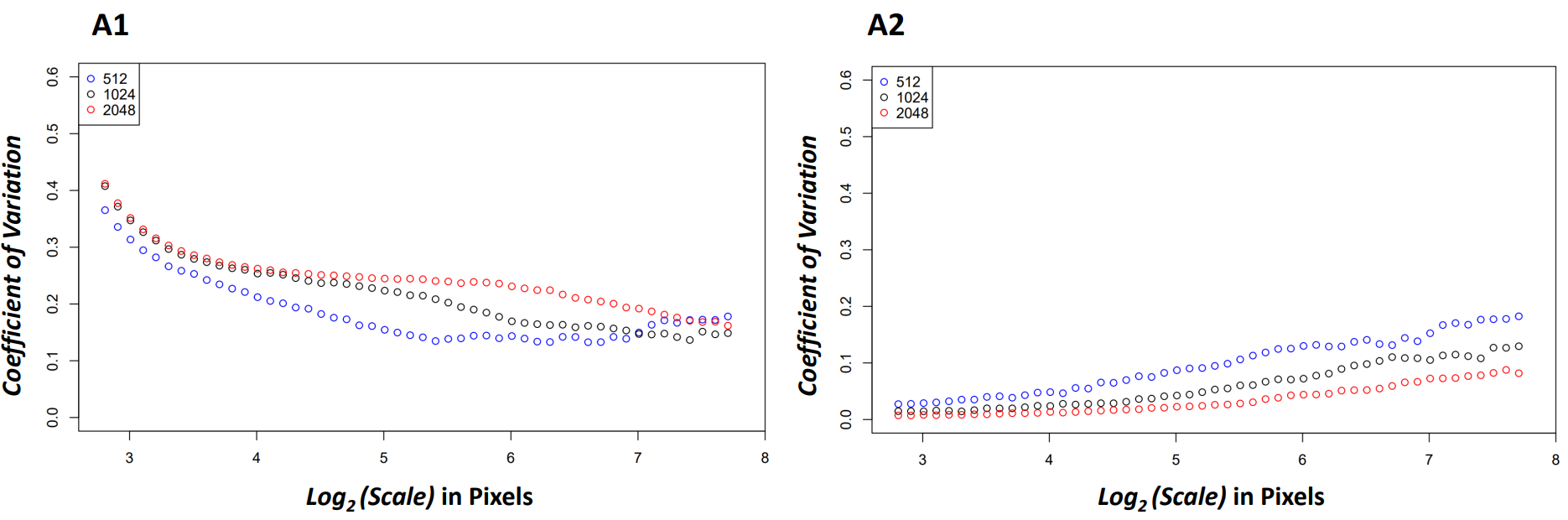


**Supplementary Figure 2**: Using white noise calibration images of dimensions 512x512 (blue), 1024x1024 (black), and 2048x2048 (red) pixels for coefficient of variation calculation. The anisotropy factor across the 50 wavelet size scales was calculated for each white noise image and then all 136 image anistropy factors were used to calculate mean and standard deviation at each size scale. Coefficent of variaiton was calculated as standard deviation divided by mean using both fixed binning anistropy factors (A1) and variable binning anistropy factors (A2).


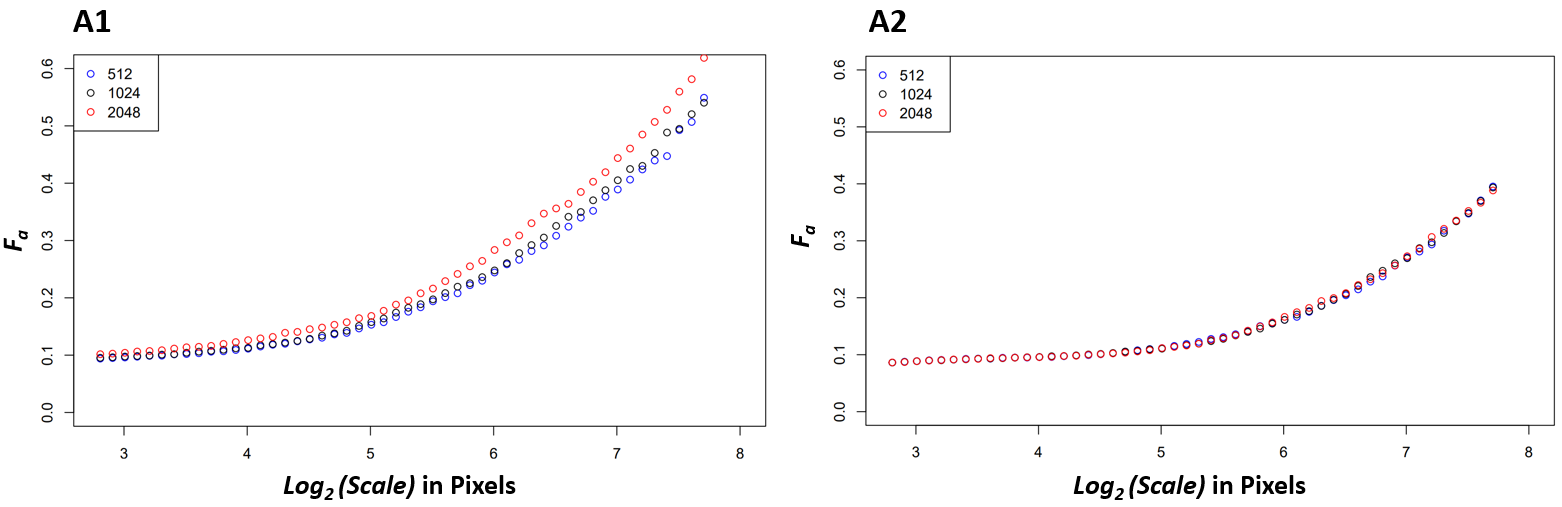


**Supplementary Figure 1**: Using Brownian and white noise calibration images of dimensions 512x512 (blue), 1024x1024 (black), and 2048x2048 (red) pixels to study size and edge effects. For size effects, the median anisotropy factor across the 50 wavelet size scales was calculated from the full image for Brownian and white noise. For edge effects, the median anisotropy factor across the 50 wavelet size scales was calculated using only the 512x512 pixel center cutout of the wavelet convolved images from the 3 dimensions for Brownian (A1) and white noise (A2).
